# Pre-procedural three-dimensional computed tomography vs. angiographic assessment for patent ductus arteriosus stenting: a comparative analysis

**DOI:** 10.3389/fcvm.2026.1767627

**Published:** 2026-03-31

**Authors:** David Hochstein, Liran Ceausu, Avshalom Shaffer, Netanel Nagar, Oliana Vajgovski, Yishay Salem, Yisrael Parmet, Shai Tejman-Yarden, Sharon Borik Chiger

**Affiliations:** 1 Internal Medicine Department C, Sheba Medical Center, Ramat Gan, Israel; 2 Engineering in Medicine Lab (EiM), Sheba Medical Center, Ramat Gan, Israel; 3 The School of Medicine, Tel Aviv University, Tel Aviv, Israel; 4 The Edmond and Lily Safra Children’s Hospital, Sheba Medical Center, Ramat Gan, Israel; 5 Department of Industrial Engineering and Management, Ben Gurion University, Beer Sheva, Israel

**Keywords:** 3-dimensional reconstruction, congenital heart disease, interventional cardiology, patent ductus arteriosus stenting, pre-procedural planning

## Abstract

**Background:**

Patent Ductus Arteriosus (PDA) stenting has become an established albeit complex intervention in ductal dependent congenital heart disease. Accurate pre-procedural planning is essential for successful stent placement, yet the optimal measurement methodology remains debated. This study compared the accuracy and clinical utility of routine pre-procedural three-dimensional (3D) computed tomography-derived measurements with traditional angiographic assessments for PDA stent sizing.

**Methods:**

We analyzed 21 consecutive PDA stenting cases from Sheba Medical Center (01/2021–10/2023) with a median age of 14 days (IQR: 8–19) and mean weight of 3.6 ± 2.2 kg. Patients were stratified by anatomical complexity: Group 1 (0 turns, *n* = 6), Group 2 (1 turn, *n* = 3), and Group 3 (≥2 turns, *n* = 12). PDA length measurements from CTA-derived 3D reconstruction and 2D angiography were compared against the actual stent length used. Statistical analysis included Pearson correlation, Bland–Altman agreement, and concordance correlation coefficients (CCC).

**Results:**

In the overall cohort, both 3D (*r* = 0.692) and angiographic (*r* = 0.811) measurements correlated with stent length. Performance varied significantly by anatomical complexity. In Group 1 (straight), angiography demonstrated favorable accuracy with lower bias (−0.28 mm vs. 1.33 mm) and higher concordance (CCC: 0.807 vs. 0.681). In Group 2 (moderate tortuosity), angiographic performance showed greater underestimation and lower concordance (Bias −5.67 mm; CCC: 0.168), while 3D remained reliable (Bias −2.33 mm; CCC: 0.526). In Group 3 (severe tortuosity), 3D overestimated length (+3.1 mm) while angiography underestimated it (−2.88 mm), though angiographic consistency was higher (CCC: 0.704 vs. 0.559).

**Conclusion:**

Pre-procedural 3D measurements provide consistent reliability across varying degrees of anatomical complexity, offering an advantage in cases with moderate tortuosity where angiography may be vulnerable to foreshortening. Angiography remains highly accurate for straightforward anatomy. A complexity-stratified imaging approach is recommended to optimize stent selection.

## Introduction

Congenital heart disease (CHD) encompasses a spectrum of structural abnormalities of the heart and great vessels present at birth, affecting approximately 1% of live births worldwide (1, 2). Among these, duct-dependent pulmonary circulation lesions represent a critical subgroup in both univentricular and biventricular physiology (e.g., pulmonary atresia, critical pulmonary stenosis, Tetralogy of Fallot). In these cases, obstruction of the outflow tract to the lungs necessitates a patent ductus arteriosus until adequate pulmonary blood flow is established for cardiac output and oxygenation. During fetal life, the ductus arteriosus serves a vital role by diverting right ventricular output away from the non-functioning fetal lungs toward the descending aorta, ensuring adequate systemic oxygen delivery from the placenta (3).

In certain subcategories of CHD, the ductus arteriosus becomes essential for maintaining adequate systemic or pulmonary blood flow. This subcategory, termed ductal-dependent CHD, includes critical lesions such as hypoplastic left heart syndrome, pulmonary valve atresia or severe stenosis, Tetralogy of Fallot, and critical aortic stenosis (4). For these patients, maintaining ductal patency is life-sustaining and is initially achieved with prostaglandin E1 infusion (5). In cases in which a temporizing measure is necessary prior to or instead of surgical repair, percutaneous PDA stenting has emerged as a pivotal intervention, largely replacing the surgical Blalock Taussig Thomas shunt (6).

Notwithstanding its growing role in neonatal and infant cardiac care, PDA stenting presents several technical challenges due to variable ductal anatomy, angulation, and the fragility of the patient population. Optimal outcomes are closely tied to precise pre-procedural planning, particularly accurate assessment of ductal dimensions to inform stent selection (7). Conventionally, stent sizing relies on angiographic measurements obtained intra-procedurally. However, this approach can prolong procedure time, bring on the use of multiple stents, increase radiation and contrast exposure, and may inadequately capture the complex three-dimensional anatomy of the PDA (8).

Recent advances in cardiac imaging, particularly three-dimensional reconstruction of computed tomography and virtual reality (VR) assessment, offer the potential for detailed anatomical assessment prior to catheterization (9). These modalities may enhance procedural planning, reduce catheterization time, and improve patient safety. Nevertheless, there is limited literature on the clinical utility and accuracy of 3D-based preplanning compared to conventional angiography in predicting optimal stent sizing.

This study compared CTA-derived 3D ductal length measurements and intra-procedural angiographic length estimates used for PDA stent sizing. The primary objective was to evaluate measurement association and agreement with implanted stent length using correlation analysis, Bland–Altman bias/limits of agreement, and Lin's concordance correlation coefficient (CCC). Potential downstream effects on procedural efficiency (fluoroscopy time, radiation dose, contrast volume, and procedural duration) were not measured in this study and remain topics for future prospective evaluation. We hypothesized that CTA-derived 3D curvilinear measurements would be particularly informative in anatomies where 2D projection is vulnerable to foreshortening.

## Methods

### Study design and population

This retrospective analysis included 21 consecutive patients who underwent PDA stenting at Sheba Medical Center between January 2021 and October 2023. All patients had routine pre-procedural 3D imaging and procedural angiography available for analysis. The study was approved by the institutional review board with waiver of informed consent due to the retrospective nature of the analysis.

Patients were stratified based on procedural complexity and clinical endpoints. In accordance with recently accepted nomenclature, patients were subdivided into three groups based on the number of turns in the PDA (10); Group 1 comprised patients with no turns (*n* = 6, 29%), Group 2 with 1 turn (*n* = 3, 14%) and group 3 with 2 or more turns (*n* = 12, 57%). Additionally, 18 (86%) patients required one stent while 3 (14%) required multiple stents. Initially, five procedures were recorded as involving >1 stent. For the present analysis, we restricted the “multiple-stent” category to cases in which two or more stents were implanted to span the ductal segment (true multi-stent ductal coverage). Two of the five cases were reclassified because the additional stent did not represent ductal length coverage (e.g., adjunctive/overlapping positioning or non-length–driven deployment) and therefore was not comparable to a “multi-stent” ductal-length strategy. One case involved branch pulmonary artery stenosis which was treated using a second stent into the contralateral narrow branch (therefore analyzed as single-stent for PDA-length comparison), and one was a stent-in-stent case in which a second stent was implanted due to narrowing caused by PDA material caught in the first stent. After this adjudication, 3 cases met criteria for true multi-stent ductal coverage, yielding 18 single-stent and 3 multiple-stent procedures for the procedural-complexity stratified analyses.

Two patients with unsuccessful PDA stent implantation were excluded form this study.

### Image acquisition and measurement protocols

Computed tomography scanning was performed using 16-channel or higher multidetector CT scanners with standardized parameters of 120 kVp and 100 mAs (Brilliance iCT; Philips Healthcare, Cleveland, OH, USA). All CT scans were ECG-gated and acquired during systole 40%–50% cardiac cycle and according to institutional protocol and reviewed by an experienced, board-certified radiologist. All images were performed within 1–2 days prior to PDA stenting under sedation with intravenous Midazolam with or without Ketamine or Fentanyl, in order to minimize motion artifacts. Prostaglandin-E infusion was discontinued or lowered to the minimal clinically accepted dose two hours prior to the exam for patient anatomy emphasis. Only axial images obtained in the supine position were analyzed to ensure consistency.

**Figure 1:**
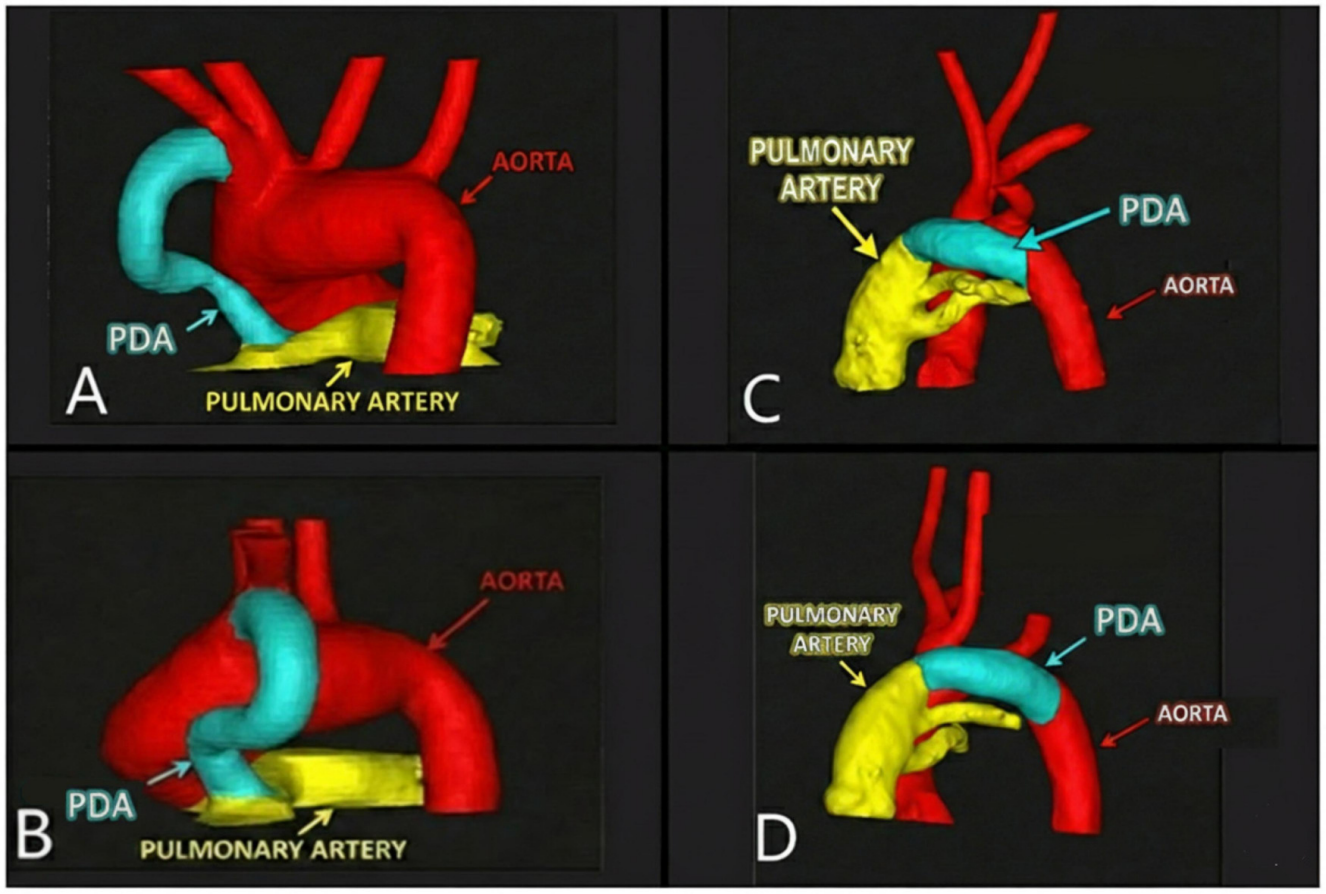
Three-dimensional reconstruction of the great vessels and patent ductus arteriosus (PDA). The aorta is depicted in red, the pulmonary artery in yellow, and the PDA in blue. Panels **(A,B)** illustrate examples of tortuous PDA morphology, while panels **(C,D)** display examples of straight PDA morphology.

**Figure 2:**
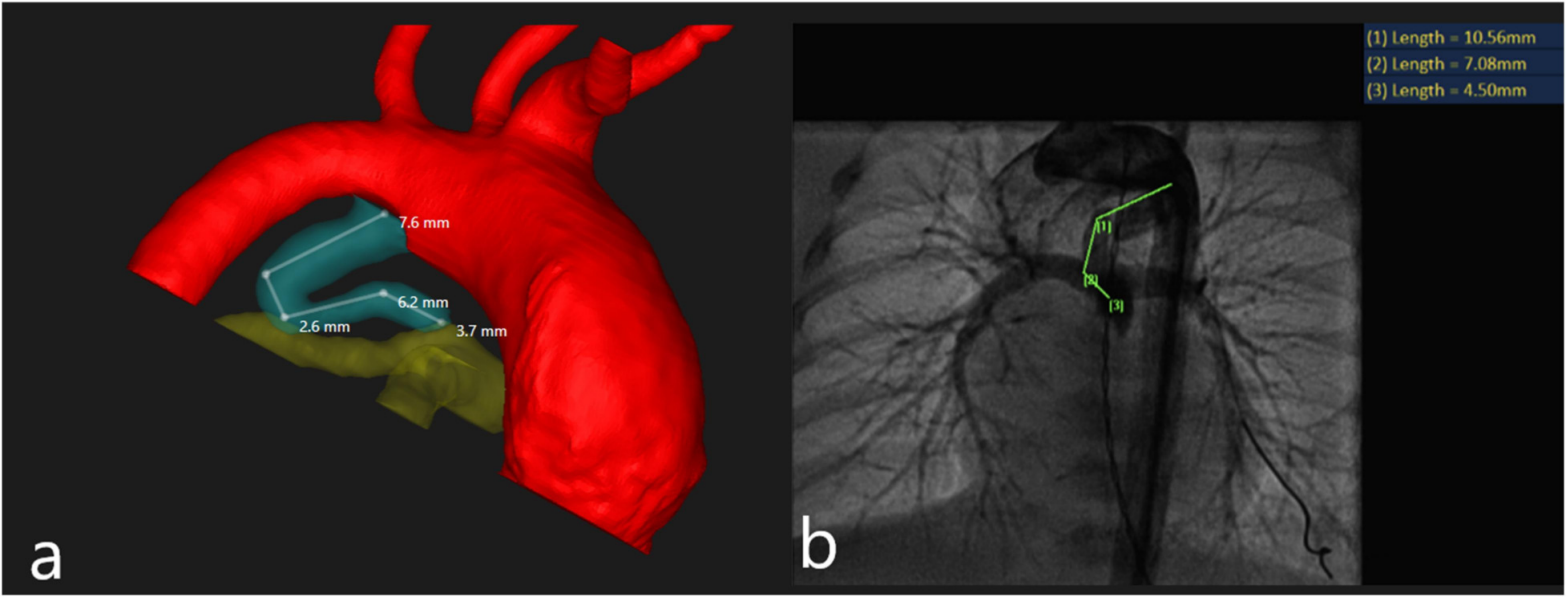
Panel **(a)** 3D reconstruction in which the aortic and pulmonary artery ductal orifices were identified on the model, and ductal length was measured along the curvilinear ductal course using a multi-point surface path tool approximating the duct centerline. Panel **(b)** angiography measurement of a tortuous patent ductus arteriosus. A multi-segment line (green) traces the length of the vessel along its tortuous path on the 2D angiographic image.

Three-dimensional model generation and CTA-derived length measurement: Three-dimensional segmented surface models were created from CTA DICOM data using D2P® software (3D Systems Inc., Littleton, CO, USA). Segmentation included the aorta, main pulmonary artery, and PDA to facilitate clear identification of the ductal origin and insertion. The duration of an individual 3D reconstruction was approximately 30–45 min per case (Figure 1). Ductal length measurements were performed on the segmented 3D surface model by imaging specialists blinded to procedural outcomes. The aortic and pulmonary artery ductal orifices were identified on the model, and ductal length was measured along the curvilinear ductal course using a multi-point surface path tool approximating the duct centerline (Figure 2a). This approach was selected to standardize landmark identification and to capture the three-dimensional geometry of tortuous ducts, where planar measurements may be sensitive to viewing angle and foreshortening and support consistent pre-procedural planning in complex anatomy. Measurements required approximately 15–30 min per patient.

Procedural angiographic images were obtained during cardiac catheterization using Phillips AlluraClarity & Siemens Artis icono biplane angiographic systems. Measurements were performed by experienced pediatric interventional cardiologists using standard system calibration techniques. PDA length was measured from the aortic orifice to the pulmonary artery insertion point using the projection that best demonstrated ductal anatomy without foreshortening (Figure 2b). Since angiography provides a 2D projected measurement while CTA-based measurements are derived from a 3D curvilinear path on a segmented model, the two modalities do not quantify an identical geometric quantity in tortuous anatomy.

The reference standard consisted of actual stent lengths used during the procedure, representing the clinical decision-making of experienced interventional cardiologists; for the 3 with multiple stents overall stent length was used considering the multiple stents placed. To reflect standard ductal stenting practice and ensure complete ductal coverage, the “planned” stent length was defined as the measured ductal length plus an allowance for 1–2 mm of stent overhang at each ductal end (i.e., proximal and distal margins) to account for landing-zone coverage, foreshortening, and minor measurement uncertainty.

### Statistical analysis

Descriptive statistics were calculated for all variables, with continuous variables presented as mean ± standard deviation (SD) or median with interquartile range (IQR) where appropriate. Categorical variables are presented as frequencies and percentages. The primary endpoints were correlation strength and measurement accuracy between 3D and angiographic measurements compared to actual stent lengths.

Correlation analysis was performed using Pearson correlation coefficients (*r*) with 95% confidence intervals. Agreement between measurement methods and actual stent lengths was assessed using Bland–Altman analysis, calculating bias (mean difference), limits of agreement (bias ± 1.96 × SD), and Lin's concordance correlation coefficient (CCC, pc). CCC values were interpreted based on established reliability scales: <0.40 indicates poor reliability, 0.40–0.59 moderate, 0.60–0.74 good, and ≥0.75 excellent reliability (11).

Demographic and anatomical characteristics were compared across the three study groups using one-way ANOVA for normally distributed continuous variables and Kruskal–Wallis tests for non-normally distributed variables. Categorical variables were compared using the Monte Carlo Fisher–Freeman–Halton exact test. Comparisons between correlation coefficients were performed using Fisher's *z*-test for independent groups and Williams' test for dependent overlapping correlations. Differences between concordance correlation coefficients (CCC) were evaluated using *Z*-tests. To account for the limited sample size in Group 2 (*n* = 3), bootstrap resampling was performed to generate distributions of the differences in CCC, providing robust confirmation of performance disparities. Statistical significance was defined as *p* < 0.05.

All analyses were performed using R version 4.4.2 (R Foundation for Statistical Computing, Vienna, Austria).

## Results

### Patient characteristics

The study cohort comprised 21 patients with a median age of 14 days (IQR: 8–19) and a mean weight of 3.6 ± 2.2 kg (Table 1). Male patients constituted 52.4% (*n* = 11) of the cohort. PDA anatomy was classified into three groups based on the number of turns: Group 1 (0 turns) consisted of 6 patients (29%), Group 2 (1 turn) included 3 patients (14%), and Group 3 (≥2 turns) comprised 12 patients (57%). Vascular access was obtained via the femoral artery in all 21 cases (100%).

**Table 1:** Baseline patient characteristics stratified by procedural complexity. Group 1 comprised patients with 0 turns in the PDA (*n* = 6), Group 2 included patients with 1 turn (*n* = 3) And Group 3 included patients with ≥2 turns. Data are presented as mean ± standard deviation for continuous variables and number (percentage) for categorical variables.

Characteristic	All patients (*n* = 21)	Group 1: 0 turns (*n* = 6)	Group 2: 1 turn (*n* = 3)	Group 3: ≥2 turns (*n* = 12)	*P* value
Demographics					
Age, days, mea*n* ± SD	57.7 ± 159.1	53.3 ± 54.6	15.7 ± 9.5	70.4 ± 209.7	
Age, days, median (range)	14 (2–736)	30 (11–150)	16 (6–25)	8 (2–736)	0.098
Weight, kg, mean ± SD	3.6 ± 2.2	4.0 ± 1.4	2.4 ± 0.7	3.7 ± 2.7	0.597
Weight, kg, median (range)	3 (1–12)	3 (2–6)	2 (1–3)	3 (2–12)	
Male gender, *n* (%)	11 (52.4)	4 (66.7)	1 (33.3)	6 (50.0)	0.713
Cardiac Anatomy					
Univentricular physiology, *n* (%)	14 (66.7)	4 (66.7)	3 (100.0)	7 (58.3)	0.802
Biventricular physiology, *n* (%)	6 (28.6)	2 (33.3)	0 (0.0)	4 (33.3)	0.802
Unspecified physiology, *n* (%)	1 (4.8)	0 (0.0)	0 (0.0)	1 (8.3)	0.802
PDA Anatomy					
Tortuous, *n* (%)	15 (71.4)	0 (0.0)	3 (100.0)	12 (100.0)	<0.001
Straight, *n* (%)	6 (28.6)	6 (100.0)	0 (0.0)	0 (0.0)	<0.001
Procedural Access					
Femoral artery, *n* (%)	21 (100.0)	6 (100.0)	3 (100.0)	12 (100.0)	1.000
PDA Measurements					
3D length, mm, mean ± SD	22.6 ± 7.3	18.2 ± 6.7	17.3 ± 2.2	26.2 ± 6.6	0.027
3D length, mm, range	11.2–36.3	11.2–26.4	15.2–19.5	17.0–36.3	
Angiographic length, mm, mean ± SD	18.3 ± 4.8	16.6 ± 2.9	14.0 ± 1.7	20.2 ± 5.2	0.074
Angiographic length, mm, range	11.0–29.0	14.1–22.0	12.0–15.0	11.0–29.0	
Stent Characteristics					
First stent length, mm, mean ± SD	20.8 ± 5.5	16.8 ± 5.2	19.7 ± 5.7	23.1 ± 4.7	0.062
Total stent length, mm, mean ± SD	23.7 ± 9.6	16.8 ± 5.2	19.7 ± 5.7	28.1 ± 9.9	0.038
Number of stents, mean ± SD	1.2 ± 0.5	1.0 ± 0.0	1.0 ± 0.0	1.3 ± 0.7	0.354
Anatomical Differences by PDA Type					
Group	3D length, mm (mean ± SD)		Angio length, mm (mean ± SD)		*P*-value
Group 1 (0 turns, *n* = 6)	18.2 ± 6.7		16.6 ± 2.9		0.463
Group 2 (1 turn, *n* = 3)	17.3 ± 2.2		14.0 ± 1.7		0.038
Group 3 (≥2 turns, *n* = 12)	26.2 ± 6.6		20.2 ± 5.2		0.001

3D, three-dimensional computed tomography; n, number; PDA, patent ductus arteriosus; SD, standard deviation.

Cardiac anatomy distribution included 14 patients (66.7%) with univentricular physiology and 6 patients (28.6%) with biventricular physiology. Mean 3D-measured PDA length was 22.6 ± 7.3 mm, while mean angiographically-measured PDA length was 18.3 ± 4.8 mm. Anatomical analysis revealed that increasing vessel complexity across the groups was associated with larger mean dimensions and a significant divergence between measurement modalities. Specifically, in Group 3, the 3D-measured length (26.2 ± 6.6 mm) was significantly longer than the angiographic measurement (20.2 ± 5.2 mm, *p* = 0.001), consistent with the foreshortening effect of 2D imaging on complex morphology.

### Overall measurement performance

In the complete cohort of 21 patients, both measurement modalities demonstrated correlations with actual stent length. Three-dimensional CT measurements achieved a Pearson correlation coefficient of *r* = 0.692, while angiographic measurements demonstrated *r* = 0.811. Direct comparison revealed no statistically significant difference between correlation coefficients (*p* = 0.178) (Table 2).

**Table 2:** Overall measurement performance (*n* = 21) comparison of 3D and angiographic measurements with actual stent length in the complete cohort.

Measurement parameter	3D (PDA length)	Angiographic (straight)	*P*-value
Pearson Correlation (*r*)	0.692	0.811	0.178
Bias (mm)	1.48	−1.16	—
Mean Square Error (mm^2^)	22.82	19.38	—
CCC	0.635	0.698	0.513

CCC, concordance correlation coefficient.

**Figure 3:**
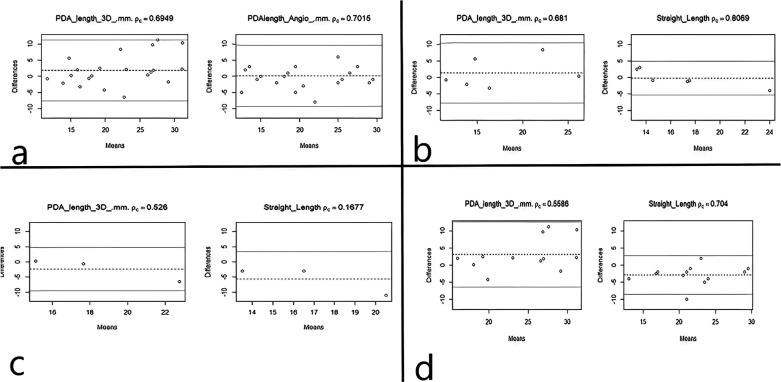
Bland–Altman agreement plots comparing CTA-derived 3D measurements and angiographic measurements with implanted stent length across tortuosity groups. Panels show the difference between each measurement method and the implanted stent length plotted against the mean of the two values. **(a)** Overall cohort (*n* = 21). **(b)** Group 1: 0 turns (*n* = 6). **(c)** Group 2: 1 turn (*n* = 3). **(d)** Group 3: ≥2 turns (*n* = 12). The solid horizontal line indicates the mean difference (bias), and dashed lines indicate the 95% limits of agreement (bias ± 1.96 SD). Agreement is summarized using Lin's concordance correlation coefficient (CCC) for each panel.

**Figure 4:**
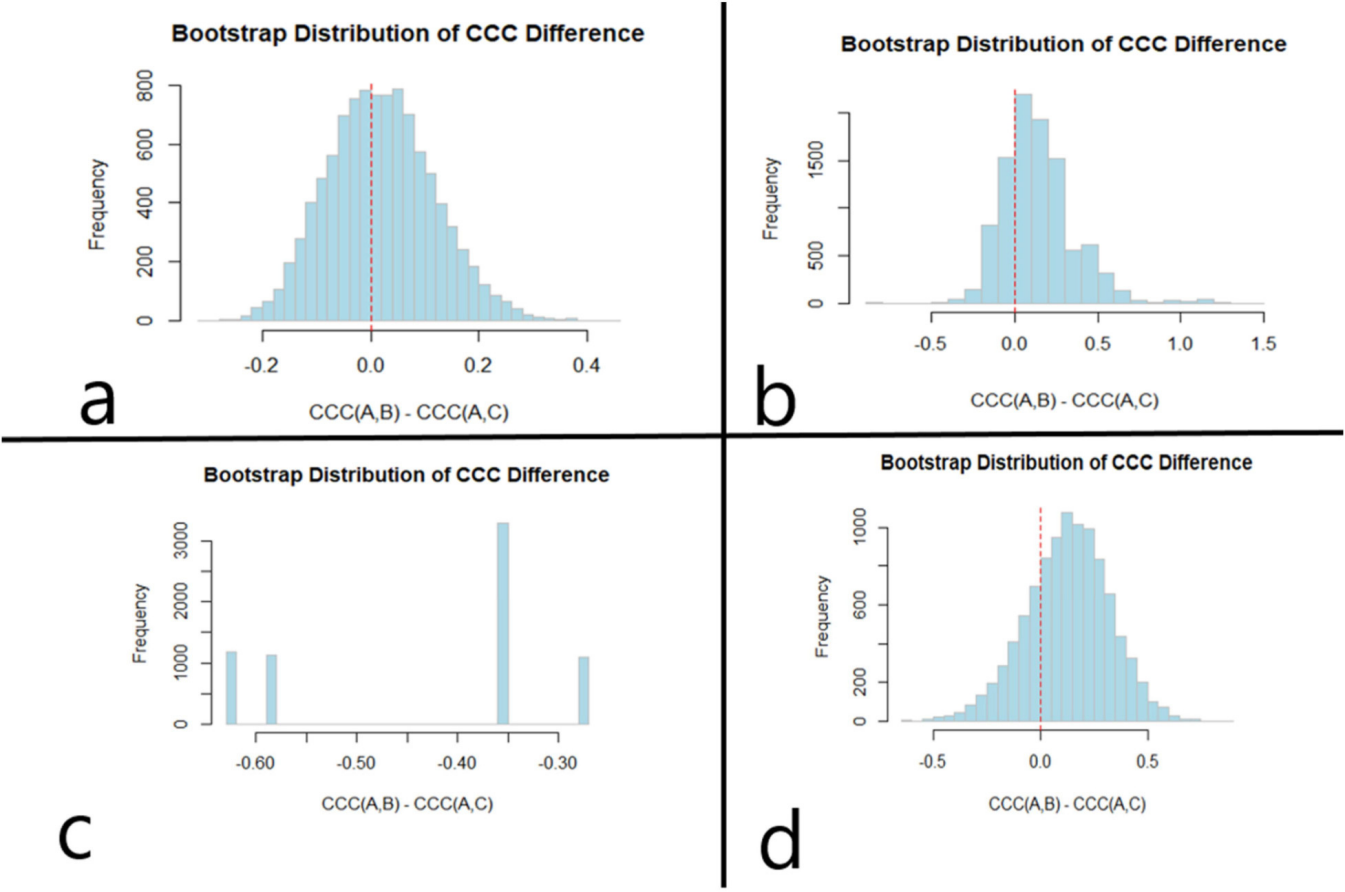
Bootstrap comparison of agreement (Lin's concordance correlation coefficient, CCC) between CTA-derived 3D and angiographic measurements across tortuosity groups. Panels display the bootstrap distributions of CCC for each modality and the corresponding between-method comparison within each stratum. **(a)** Overall cohort (*n* = 21). **(b)** Group 1: 0 turns (*n* = 6). **(c)** Group 2: 1 turn (*n* = 3). **(d)** Group 3: ≥2 turns (*n* = 12). *P*-values reflect the bootstrap-based comparison of CCC between modalities within each panel.

Bias analysis demonstrated differential accuracy patterns between modalities. Three-dimensional CT measurements showed an overestimation bias of 1.48 mm [Mean Square Error (MSE): 22.82 mm^2^], while angiographic measurements demonstrated a bias of −1.16 mm (MSE: 19.38 mm^2^) (Table 2). Concordance correlation coefficient (CCC) analysis revealed comparable agreement for both methods (3D: CCC = 0.635; angiographic: CCC = 0.698; *p* = 0.513) (Table 2, Figures 3a, 4b).

### Stratified analysis by procedural complexity

#### Group 1 (0 turns)

Among 6 patients in Group 1, both measurement methods maintained strong correlations with stent length (Table 3). Angiographic measurements demonstrated a particularly high correlation of *r* = 0.952 compared to *r* = 0.722 for 3D measurements (Tables 3, 4). Accuracy assessment favored the angiographic method in this group, showing a minimal bias of −0.28 mm and a significantly lower MSE of 5.79 mm^2^ compared to 1.33 mm and 19.73 mm^2^ for 3D (Table 3). Agreement analysis using CCC demonstrated high reliability for the angiographic method (pc = 0.807) over 3D (pc = 0.681) (Table 3). This relationship is depicted in the group-specific Bland–Altman plot (Figure 3b) and bootstrap analysis (Figure 4b).

**Table 3:** Stratified analysis by PDA morphology. Comparison of measurement performance between the different PDA morphology groups.

Parameter	Group 1 (*n* = 6)	Group 2 (*n* = 3)	Group 3 (*n* = 12)
3D (PDA Length)			
Pearson Correlation (*r*)	0.722	0.971	0.686
Bias (mm)	1.33	−2.33	3.1
Mean Square Error (mm^2^)	19.73	14.26	30.96
CCC	0.681	0.526	0.559
Angiographic (Straight)			
Pearson Correlation (*r*)	0.952	0.711	0.837
Bias (mm)	−0.28	−5.67	−2.88
Mean Square Error (mm^2^)	5.79	46.33	15.85
CCC	0.807	0.168	0.704

**Table 4:** Correlation matrix analysis pearson correlation coefficients between measurement methods and actual stent length by group values in parentheses represent statistical significance of individual correlations. Correlation comparison uses overlapping correlation comparison tests (Williams, Steiger, meng methods)*.*

Group	3D vs. stent length	Angiographic vs. stent length	3D vs. angiographic	Correlation comparison *P*-value
Group 1 (*n* = 6)	0.722 (*p* = 0.105)	0.952 (*p* = 0.003)	0.744 (*p* = 0.090)	0.180 (Williams)
Group 2 (*n* = 3)	0.971 (*p* = NA)	0.711 (*p* = NA)	0.859 (*p* = NA)	1.000 (Dunn)
Group 3 (*n* = 12)	0.686 (*p* = 0.014)	0.837 (*p* < 0.001)	0.675 (*p* = 0.016)	0.320 (Williams)

#### Group 2 (1 turn)

In the 3 patients in Group 2, 3D measurements maintained a high correlation (*r* = 0.971) with the first stent length, while angiographic correlation was lower (*r* = 0.711) (Tables 3, 4). This group showed the most significant performance divergence; while 3D bias was −2.33 mm (MSE: 14.26 $mm), angiographic measurements showed a substantial underestimation bias of −5.67 mm and a high MSE of 46.33 mm^2^ (Table 3). Agreement analysis reflected this disparity, with 3D (pc = 0.526) outperforming angiography (pc = 0.168) (Table 3). The deviation in angiographic performance is evident in the Bland–Altman plot (Figure 3c) and the significant difference found in bootstrap analysis (*p* < 0.001) (Figure 4c), yet the sample size is small.

#### Group 3 (≥2 turns)

For the 12 patients in Group 3, both methods showed moderate correlation with stent length (3D: *r* = 0.686; Angio: *r* = 0.837) (Tables 3, 4). Accuracy analysis revealed a systematic underestimation by angiography (bias: −2.88 mm) and an overestimation by 3D (bias: 3.10 mm) (Table 3). Agreement analysis indicated that while the angiographic method (pc = 0.704$) remained reliable, 3D measurements (pc = 0.559$) provided a viable alternative for these complex cases (Table 3). These findings are illustrated in (Figure 3d) and (Figure 4d).

### Performance consistency across procedural complexity

Comparative analysis showed that CTA-derived 3D agreement was relatively consistent across tortuosity strata, with CCC point estimates ranging from 0.526 to 0.681 across Groups 1–3, whereas angiographic agreement varied more widely (CCC: 0.168–0.807), driven primarily by the low CCC point estimate in Group 2 (*n* = 3) (Tables 3, 5). Between-group CCC comparisons demonstrated a significant difference for angiography between Groups 1 and 2 (*p* = 0.006), while 3D CCC did not differ significantly between these groups (*p* = 0.690) (Table 5). Given the small sample size in Group 2, subgroup findings should be interpreted as exploratory.

**Table 5:** Between-group performance comparison statistical comparison of measurement performance between the different PDA morphology groups; statistical tests used: fisher's *z*-test for correlations, *Z*-test for CCC comparisons.

Comparison	Test statistic	*P*-value	Clinical Interpretation
3D correlation: Group 1 vs. 2	*z* = −0.000	1	No significant difference
Angiographic correlation: Group 1 vs. 2	*z* = 0.000	1	No significant difference
3D CCC: Group 1 vs. 2	*z* = 0.398	0.69	No significant difference
Angiographic CCC: Group 1 vs. 2	*z* = 2.773	0.006	Performance degradation
3D CCC: Group 2 vs. 3	*z* = −0.095	0.924	No significant difference
Angiographic CCC: Group 2 vs. 3	*z* = −1.929	0.054	Marginal/No significant difference

Bootstrap comparison analysis confirmed that while both methods performed comparably in the overall cohort (*p* = 0.878) (Figure 4a), a statistically significant difference in measurement reliability emerged specifically in Group 2 (*p* = 0.000) (Figure 4c). No significant difference in measurement reliability was observed in Group 1 (*p* = 0.513) (Figure 4b) or Group 3 (*p* = 0.49) (Figure 4d). As mentioned previously, group 2 represented a small sample size and therefore should be interpreted accordingly.

## Discussion

This study provides a systematic comparison of 3D vs. 2D angiographic measurements for PDA stent sizing, demonstrating complementary performance characteristics that support case-specific measurement selection. Our findings corroborate and extend previous evidence regarding the utility of 3D imaging approaches while revealing important performance differences based on procedural complexity.

PDA quantification is inherently challenging and historically dependent on the quality of vascular access and the specific angiographic projection obtained (6). In our cohort, vascular access was uniformly obtained via the femoral artery (100%), utilizing a cut pigtail catheter technique in most cases (Table 1). While this standardized approach avoids the morbidity of additional arterial access, it inherently limits the operator to specific angiographic views that may not perfectly profile the ductal length, particularly in tortuous anatomy (12, 13).

An advantage of 3D reconstruction observed in this study is the enhanced definition of anatomical landmarks. Unlike 2D angiography, where the start and finish of the ductus can be obscured by contrast overlap or suboptimal imaging, 3D segmentation allows for a clearer assessment of the PDA origin and insertion (9, 14). This capability reduces ambiguity regarding the native anatomy and may mitigate the overestimation of anatomy sometimes seen when angiographic projections are not perfectly orthogonal to the vessel axis.

Analysis of our cohort indicated that while angiographic measurements excelled in straightforward cases, 3D reconstruction offered a more consistent reliability profile across varying degrees of anatomical complexity. In patients with straight ductal anatomy (Group 1), angiographic measurements demonstrated high accuracy with lower bias (−0.28 mm vs. 1.33 mm) and strong concordance (CCC = 0.807). This suggests that for simple anatomy, standard 2D angiography remains a highly effective tool, benefiting from real-time visualization without the need for complex post-processing (15).

However, a notable performance divergence emerged in cases with moderate tortuosity (Group 2). Here, angiographic reliability showed a decline (CCC = 0.168), accompanied by an underestimation bias of −5.67 mm, likely due to foreshortening of the vessel curve in 2D projection. In contrast, 3D measurements in this group maintained a stronger correlation (*r* = 0.971) and stable concordance (CCC = 0.526). This supports the utility of 3D planning in detecting vessel length that 2D projections may obscure, offering a consistent baseline for sizing when angiographic profiling is limited (16).

An intriguing finding emerged in the most complex group (Group 3, ≥2 turns). While 3D imaging provided a comprehensive anatomical assessment, it demonstrated a systematic overestimation of stent length (+3.10 mm), whereas angiography continued to underestimate (−2.88 mm) but achieved higher statistical consistency (CCC = 0.704). We hypothesize that this reflects a geometry–device interaction. The 3D measurements quantify the native curvilinear ductal course, essentially the “longest path” (17). However, the rigid stent likely straightens the vessel upon deployment, following a path significantly shorter than the native centerline. Consequently, while 3D provides the most accurate measurement of the *native anatomy* without overestimation of the structure itself, the *stent path* effectively bypasses the tortuosity. Angiographic foreshortening, by coincidence, may sometimes align closer to this final “straightened” stent length. This suggests that optimal planning in severe tortuosity may require an algorithmic adjustment subtracting a “straightening factor” from the accurate 3D centerline to predict the final device configuration.

Our findings are corroborated by convergent evidence from multiple centers using 3D imaging approaches. Jadhav et al. (17) demonstrated that angiography consistently underestimated ductal length by 3.2 ± 1.6 mm and tortuosity index by 14.8 ± 7.2 compared to 3D models in a cohort of 12 patients undergoing ductal stenting. Their study further demonstrated that 3D planning enabled successful procedural planning in 100% of attempted cases, with accurate prediction of optimal vascular access approach and identification of patients unsuitable for ductal stenting due to anatomical complexity.

Similarly, Chamberlain et al. (18) reported their experience with 3D angiography and modeling in 12 patients, demonstrating that angiography consistently underestimated ductal length (−3.2mm ± 1.6 mm) and tortuosity (−14.8 ± 7.2), findings that align precisely with the Jadhav study. Their 3D planning approach enabled successful patient selection, with two patients being appropriately excluded from ductal stenting based on 3D assessment—one due to extreme tortuosity and another due to risk of pulmonary artery isolation. Among the nine patients who proceeded with stenting after 3D planning, there were no major procedural complications, compared to a 40% major complication rate in their historical cohort without 3D planning.

This convergent evidence from multiple centers strengthens the case for complexity-based measurement selection and validates our findings regarding the complementary roles of different assessment approaches.

### Study limitations

Several limitations warrant acknowledgment. CTA and angiography estimate ductal length using different geometric constructs (3D curvilinear path on a segmented surface model vs. 2D projected measurement), and some observed differences in bias and agreement may therefore reflect measurement geometry rather than modality performance alone. Additionally, the CTA workflow in this study relied on segmented 3D surface models, which requires dedicated software and operator expertise and may limit generalizability to centers using routine double-oblique multiplanar reformats alone.

The sample size, particularly for the moderate tortuosity subgroup (Group 2, *n* = 3), is underpowered and should be interpreted cautiously. The retrospective design introduces potential selection bias, although consecutive case inclusion was employed to mitigate this concern. Measurements were performed by experienced cardiovascular imaging specialists and interventional cardiologists; however, inter-observer variability was not formally assessed, which may influence the generalizability of our findings across operators with varying experience levels. Furthermore, the study utilized actual deployed stent length as the reference standard. While this reflects the practical clinical decision-making of experienced operators, it may not necessarily represent a theoretical “optimal” stent length in every instance. Furthermore, the subgroup analysis for Group 2 was constrained by a small sample size (*n* = 3). This lack of statistical power necessitates cautious interpretation of the observed discrepancy between measurement methods, as the dataset may be insufficient to distinguish true anatomical variance from sampling artifacts. Finally, these findings represent a single-center experience; validation across multiple centers with varying imaging protocols and operator expertise is necessary to strengthen the evidence base.

## Conclusions

This study demonstrates that measurement should be tailored to anatomical and procedural complexity for optimal PDA stent planning. 2D Angiographic measurements provide favorable accuracy and reliability for straight PDA's and single-stent procedures, while 3D measurements offer a standardized framework to define ductal endpoints and capture the true curvilinear course, and may provide added value when 2D projection is vulnerable to foreshortening or landmark ambiguity (17, 18). These findings align with recent multi-center evidence showing the complementary roles of different imaging modalities and the importance of case-specific measurement selection for optimal clinical outcomes rather than implying uniform superiority (10, 19).

We believe that centers performing PDA stenting may consider tiered measurement protocols: strictly angiographic assessment for straightforward cases (Group 1) and additional 3D-based planning for cases with moderate-to-severe tortuosity (Groups 2 and 3) or those anticipated to require multiple stents (20). Since many common workflows currently incorporate pre-procedural CTA imaging as a routine part of procedural planning prior to PDA stenting, segmentation-based 3D measurement may be a feasible adjunct in selected cases. However, small subgroups, specifically moderately tortuous and multiple stent cases remain underpowered to provide definitive, reproducible inference.

Future studies should evaluate clinical outcomes comparing complexity-stratified vs. universal measurement approaches and explore integration with emerging technologies such as virtual reality modeling for enhanced procedural planning (20). Specifically, research should aim to quantify the impact of 3D planning on procedural efficiency endpoints, including total fluoroscopy time, radiation dose, contrast volume, and overall procedural duration (18, 21).

## Data Availability

The original contributions presented in the study are included in the article/Supplementary Material, further inquiries can be directed to the corresponding author.
